# Evaluating a global classroom initiative to teach machine learning applications in healthcare

**DOI:** 10.1186/s12909-025-07918-w

**Published:** 2025-10-21

**Authors:** Sathana Dushyanthen, Frances Hooley, Kayley Lyons, Jon Parkinson, Peter Trimbel, Mike Conway, David L. Kok, Alan Davies, Angela C. Davies

**Affiliations:** 1 Centre for Digital Transformation of Health, University of Melbourne, Melbourne, VIC Australia; 2 School of Health Sciences, University of Manchester, Manchester, UK; 3 School of Computing and Information Systems, University of Melbourne, Victoria, Australia; 4 Peter MacCallum Cancer Centre, Melbourne, VIC Australia; 5 Education Development Team, University of Manchester, Manchester, United Kingdom

**Keywords:** Collaborative online international learning, Continuous professional development, Health professions education, Interprofessional, Workforce development, Evaluation, Artificial intelligence, Digital transformation, Digital health education

## Abstract

**Background:**

Global classrooms transcend geographical boundaries, fostering collaboration and knowledge exchange among learners worldwide. Artificial intelligence (AI) has the potential to enhance patient care, streamline processes, and revolutionise healthcare capabilities. However, there is a shortage of a skilled workforce capable of utilising insights to design innovative solutions. Therefore, tailored education that leverages collaborative learning, knowledge and experience sharing amongst international colleagues, may be effective in progressing digital transformation efforts.

**Objectives:**

This study explores learners’ experiences and perspectives from a 4-week online global classroom education programme between University of Melbourne, Australia and Manchester University, UK, designed to develop the AI skills of healthcare professionals. The evaluation aimed to assess the benefits, barriers, and opportunities for improving international interprofessional collaborative learning, as well as providing insights for educators.

**Methods:**

We developed a fully online short course for interprofessional healthcare professionals. In a flipped classroom model, learners (*N* = 21) completed 2 h of pre-class online learning followed by 2 h of live interactive weekly Zoom workshops for 4 weeks. Throughout the course, learners engaged in small group work, contributed their unique expertise, listened to experts in the field, and received feedback on their project pitches from an expert panel.

To evaluate the programme’s utility, a mixed methods approach was used, including pre- and post-surveys with rating scales. Learners also completed self-efficacy measures (*N* = 18), with scales mapped to specific capability statements. Weekly surveys with free-text responses provided additional feedback on course continuity and suggested improvements.

**Results:**

The self-efficacy component revealed a significant increase (*P* <.0001) in perceived confidence across all capability statements from pre- to post-course. The programme effectively provided learners with access to global perspectives from instructors, expert panels, and diverse participant experiences, offering a solid foundation to develop and refine project ideas. The final pitchathon was useful in applying the learnings. Learners reported intentions to apply knowledge to improve service delivery, develop predictive models, and collaborate with data scientists. Key recommendations include tailoring more specific, personalised learning pathways, including additional case studies, providing opportunities or deeper peer and expert interactions and fostering post-course community building.

**Conclusions:**

The global classroom facilitates learning and problem-sharing among healthcare professionals, promoting broader thinking, encouraging collaboration across diverse perspectives, and enabling a better understanding of the initiatives colleagues worldwide are undertaking. To enhance this experience, further efforts should focus on enabling more meaningful collaboration among learners during and after the course, to develop global communities of practice.

**Supplementary Information:**

The online version contains supplementary material available at 10.1186/s12909-025-07918-w.

## Background

In an era defined by rapid technological advancements, the intersection of artificial intelligence (AI) and healthcare has emerged as a pivotal frontier, promising transformative innovations to enhance patient care, streamline operational processes, and revolutionise medical capabilities and practices [1, 2]. However, the adoption of these technologies requires more than technical tools; it demands a skilled interprofessional workforce with an understanding of the importance of interprofessional collaboration, one that is capable of interpreting data, addressing ethical and governance concerns, and applying AI and machine learning (ML) in real-world contexts [1, 3]. Despite their potential, barriers such as data access, data quality issues, and a lack of comprehensive training, hinder widespread AI adoption in healthcare [1, 4]. Recognising the global significance of this intersection, educational initiatives have surfaced to equip the interprofessional healthcare workforce with the requisite skills and knowledge to harness AI’s potential [1].

Global classrooms are a form of collaborative online international learning (COIL) and represent a transformative approach to education that transcends geographical boundaries, fostering co-learning, collaboration, knowledge exchange and sharing and co-creation amongst its learners worldwide [5]. Through COIL, “students enrich their intercultural learning and critical thinking by examining a topic or problem through a cross-cultural and global lens” [6]. These learners are often from multiple organisations, professional backgrounds and healthcare contexts [5]. The course followed the COIL framework by providing a practical and applied way for different institutions to collaborate and share learning on digital healthcare education. More importantly, the learners in the UK and Australia were able to draw on shared experiences, frame local issues against global challenges and gain new perspectives and solutions. By “using a local-to-global approach” the course helped build connections across the world. The course was enriched even further by involving members from the UK Topol Fellows Programme in one of the sessions. These are a diverse mix of healthcare professionals, actively developing their digital transformation projects. The session also involved a panel of subject matter experts from academia and local healthcare institutions. The global classroom learners could reflect on real world projects, learn from insights from a range of perspectives to help address similar challenges in their own practice. By enabling participants to address shared challenges through pooling of diverse expertise, perspectives and experiences, these initiatives hold the potential to enrich learning experiences and advance global healthcare practices [7]. Over the last few years, the rapid transition to online delivery during COVID-19 and large-scale role out of web conferencing technologies across higher education institutions has made the delivery of global classrooms much more accessible to many educators and learners [8]. Embracing digital technologies, these virtual classrooms create a rich and dynamic space where diverse learners can engage in real-time discussions, and work together to address global health challenges, fostering cross-border partnerships [7, 8].

The University of Manchester (United Kingdom) and the University of Melbourne (Australia) developed and piloted a short course *Application of Machine Learning in Healthcare* in September-October 2023 focused on the application of machine learning (ML) in healthcare. It aimed to equip learners to critically evaluate ML models, and to become conversant with concepts and methods, in order to work with data science colleagues to trial AI-enabled solutions effectively and ethically in healthcare. The skills taught in the course were based on key findings from significant reviews of the healthcare workforce, such as the Topol Report [3] or the Goldacre Review [9] in the UK. These reviews emphasise the importance of upskilling healthcare professionals to effectively use AI and ML for diagnostics, patient monitoring, and personalised care, and the need for training to interpret AI-driven insights for better clinical decision-making in a digitally enabled healthcare environment.

However, given the limited time, resources, and financial support available for staff to develop foundational data science skills, training initiatives must be flexible, inclusive, and practical, allowing learners to practice working with data and to develop digital skills in safe, low-risk settings. Additionally, essential skills like interdisciplinary collaboration, problem-solving, communication, and self-efficacy are crucial in building confidence and agency. Recognising that contemporary healthcare challenges transcend geographical boundaries, international collaborative learning, and network-building for both learners and tutors, became an added outcome for the course. However, there has been a paucity of research evaluating the efficacy and impact of educational interventions centred around global classrooms and international education collaborations.

This study aims to evaluate the experiences of participants learning in a global collaborative learning environment, as well as the effectiveness and challenges of delivering interprofessional machine learning education; providing insights that will shape pedagogical strategies and potentially influence the future of healthcare education. We aim to describe the implementation, feasibility, acceptance, and outcomes of a programme to foster machine learning skills in the healthcare workforce through flipped classroom coursework, interactive workshops, international expert guidance and collaborative learning. This paper will also appraise practical considerations, including the logistics of collaboration within a global classroom initiative, with educators located in two different countries (UK and Australia), spanning vastly different time zones.

### Research questions

1. What are learners’ reactions to the global classroom model as a means of fostering international collaboration and interdisciplinary learning?
2. What benefits and barriers are there in participating and implementing an interprofessional global classroom, and what are the recommendations for improving future iterations?
3. How do learners perceive the relevance, engagement, and applicability of the course content in their individual contexts?

### Research objectives

1. Examine the global classroom model: Investigate how the global classroom approach supports interdisciplinary learning, collaboration, and international networking.
2. Gather feedback for improvement: Identify the key benefits and barriers experienced by learners and collate their recommendations for enhancing course design, delivery, and impact.
3. Evaluate learner reactions: Assess learners’ perceptions of the course content, including its relevance, engagement, and applicability to their professional contexts.

## Methods

### Programme background

The Application of Machine Learning in Healthcare short course was created through a collaboration between The University of Manchester’s School of Health Sciences and The University of Melbourne’s Centre for Digital Transformation of Health, through an international seed funding grant to support collaboration in cancer sciences. The course was a pilot for 21 successful learners, selected through an open Expression of Interest (EOI) process (over 400 applicants), consisting of 10 participants from each institution.

**Fig. 1:**
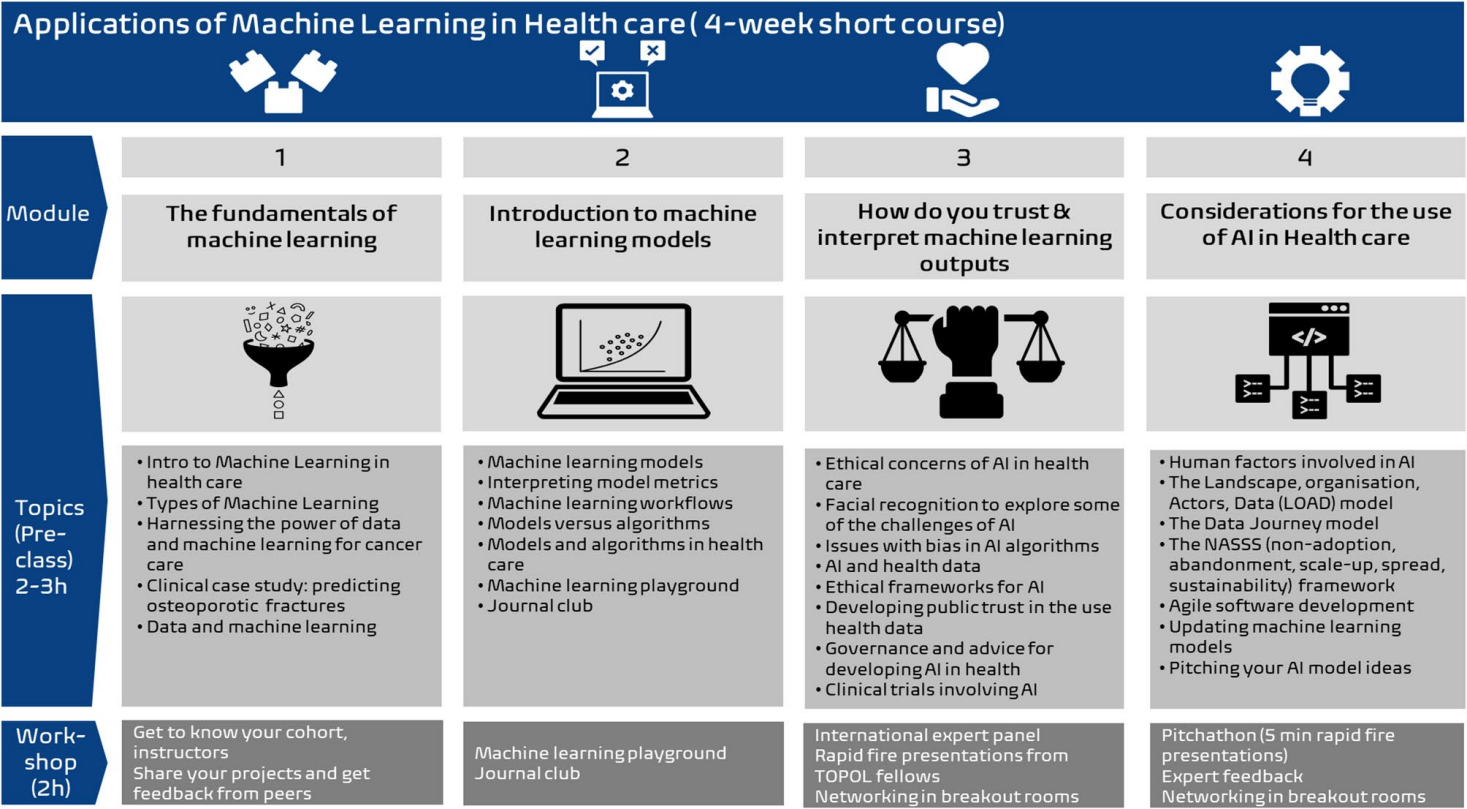
Overview of the Applied Machine Learning in Healthcare course

The short course involved a 4-week online course focussed on machine learning principles and was delivered entirely online, by a team of educators with diverse backgrounds. The course leveraged a flipped classroom learning format, including 2 h of weekly individual asynchronous pre-class learning, followed by 2 h of weekly synchronous workshops. Learners were from a range of backgrounds, including working professionals in healthcare, healthcare technology and translational research, but all had roles related to cancer sciences. Each week learners worked through activities that built on the pre-class learning, often working in breakout rooms with their inter-professional peers, sharing details of ideas for their individual projects. Learners also took part in a machine learning playground activity, journal club and a formal panel discussion with machine learning in healthcare experts. In the latter event they joined the Topol Digital Fellows, which was a shared event with the UK Topol Digital Fellowship programme [10]. In the final synchronous session, the learners had the opportunity to pitch an idea to use machine learning in their practice, to a panel of experts, from whom they received feedback (referred to as the “pitchathon”). The content of each week was split into 4 themes including: Fundamentals of machine learning; Introduction to machine learning models; trust and interpretation of machine learning outputs and Considerations for the use of AI in healthcare (Fig. 1).

### The virtual learning environment

The course was delivered on the University of Manchester’s eLab platform [11] which is a highly customisable digital workbench designed for teaching and research. It surpasses traditional virtual learning environments by providing support for multiple programming languages, enabling team-based learning through wikis and group spaces, and running code workbooks. We utilised this platform as a secure learning environment for learners. We hosted the didactic learning materials through Articulate and these learning modules were linked in eLab. Additionally, we developed Flask apps for the machine learning playground. These were able to be easily run through eLab, without needing to undertake any coding for users to access the tool.

### Learning objectives and professional capabilities

To establish a connection to professional capabilities, the course learning objectives were mapped to relevant capability statements in the NHS England Artificial Intelligence (AI) and Digital Healthcare Technologies Capability framework [4]. The framework was designed collaboratively with representatives from the University of Manchester, NHS England, healthcare professionals and industry representatives. The framework consists of six main domains: (1) Digital implementation, (2) Digital health for patients and the public, (3) Ethical, legal and regulatory considerations, (4) Human factors, (5) Health data management, (6) Artificial Intelligence. The capabilities are categorised into four levels, progressing from lower-order thinking skills and knowledge levels to higher-order ones, aligned with Bloom’s digital taxonomy. The learning objectives were mapped to the framework according to Table 1.

**Table 1:** Mapping of learning objectives against the NHS England artificial intelligence and digital health technology capability framework [4]

Learning objectives	Capability statements
Differentiate between the branches of AI and the terminology associated with machine learning	“I can define Artificial Intelligence (AI) and related sub-domains (e.g. machine learning, deep learning)”
Compare the core concepts and methodologies used in the field of machine learning (e.g. data science, statistics, mathematics, computer programming)	“I am familiar with core concepts and methodologies used in the field of Machine Learning (ML) (for example, data science, statistics, mathematics, computer programming)”
Investigate potential challenges to the successful adoption of AI in clinical practice (e.g. accessing data, data quality, ethics and governance)	“I champion a culture of ethical responsibility around the use of Artificial Intelligence (AI) and digital technology to ensure that systems and processes are fair, transparent, equitable and non-discriminatory to patients, staff and the wider public; espousing the principles of beneficence, non-maleficence and autonomy.”
Describe how an AI system can be used in a professional context	“When evaluating an AI system for use in my professional workflow, I can compare its performance against the expected standards in my professional area of practice”
Interpret AI model outputs and applications in clinical contexts	“ I am aware of how machine learning models are trained and validated with data (e.g. supervised, unsupervised, reinforcement learning, cross-validation)”
Explore how to successfully apply for funding for translation AI projects	“I can identify the contribution that AI can make to healthcare processes and how it can help me in my role, including its benefits and limitations”

### Evaluation framework

We utilised the Kirkpatrick Model of Evaluation [12] to map out our measurements (Table 2). This model is a widely used evaluation framework in education and is employed to shift researchers away from simply measuring perceptions and satisfaction. We explored evidence of whether learners changed their attitudes, knowledge, behaviour, and professional practice. Additionally, we applied a convergent parallel mixed methods approach [13] that included pre-survey, post-survey and weekly surveys. This need for consent to participate was waived and approved by the University of Melbourne Ethics Committee (Project ID: 27753) and The University of Manchester Ethics Committee (Project ID 18104). Completion of the evaluation surveys was a mandatory component of receiving the scholarship to attend the pilot course. Consent to participate declaration: this was an *opt out* ethics for all participants, therefore the participants did not need to complete the surveys if they did not want to participate in the pilot programme.

Selection for the pilot was based on ability to participate fully in the evaluation. This sample size (*N* = 21) was chosen because the pilot was working across different time zones to bring people together in the live workshops and group activities. This size enabled testing of manageability and feasibility, in order to optimise the program before offering it to wider audiences. In order to avoid EOI selection bias, a strict selection criterion was used, with two independent reviewers - one from the UK and one from Australia:

1. Meets the course pre-requisites and target audience
  Not an expert in AI
  Cancer clinician or researcher
  UK (University of Manchester or Christie Hospital only)/Australia (Victorian Comprehensive Cancer Centre only)
2. Select for diversity in the cohort
  Diversity in their current level of involvement in AI
  Diversity across industry
  Diversity across job titles
  Diversity across organisations
3. Then, break ties with the quality of the “why they want to take the course” response (e.g., rated 1-5)

**Table 2:** Application of the Kirkpatrick model of evaluation (amended by barr et al.) to this project [12]

Level	Details	Evaluation Measures and Data Sources in this Project
1	Perception of training by subjects	Pre-, weekly, and post-surveys
2a	Change of attitudes of subjects	Pre-, post- change in confidence
2b	Change of knowledge and/or skills of subjects	Pre-, post-self-efficacy changes in specific concepts (skills)
3	Changes of behaviour of subjects	Post-course post-surveys (Not assessed long-term)
4a	Change in professional practice	Post-course surveys (Not assessed long-term)
4b	Changes in patients’ condition	Not applicable

#### Pre-course and post-course surveys

The pre- and post-surveys incorporated a combination of psychological scales and open-ended questions that were self-developed, based on commonly asked survey questions for education evaluation. These questions have been iterated and improved over time, across multiple cohorts of learners in our education programs. The learners were asked to provide self-rate their knowledge of machine learning, to rate educational activities in relation to their personal career development and current role, as well as asking what we should keep, change or improve and to see how the course challenged and motivated their next development activity.

The pre- and post-survey included the same self-efficacy scale (100 points; cannot do at all to highly certain can do) [14] which has significant evidence of reliability and validity. We chose to evaluate self-efficacy as it is one of the strongest proxy measures in education to predict actual and future performance, which are more difficult and take longer to measure [15]. The ten items on the self-efficacy scale were adapted from the material taught in the course and language from the literature (e.g., use machine learning algorithms to create a model for predicting a health outcome). The open-ended questions included demographic questions (e.g., job title) and questions related to digital health identity development, course benefits, course barriers, course improvements and other suggestions or comments (Supplementary Fig. 1,2).

Surveys were designed and distributed via Qualtrics^®^. Learners were invited to complete the surveys through emails and the Learning Management System (University of Manchester’s eLab [11]). Responses to open-ended survey questions were also analysed through qualitative content analysis. An initial coding framework was developed deductively, informed by the study’s research questions and survey prompts. This included broad categories such as *perceived value of course components*, *barriers to learning*, *suggestions for improvement*, and *planned application of learning*. Additional inductive (emergent) codes were generated through iterative reading of the data to capture unanticipated themes or nuanced insights. A coder coded the text responses. The coder then solidified themes and categories under each research question. Through this iterative process, codes were grouped into higher-order themes, and representative quotes were identified to illustrate key findings. The self-efficacy scales were analysed using two tailed, paired t-test using Graph Pad Prism, to determine whether there was an improvement in self-efficacy for each of the capability statements before and after the course.

#### Weekly surveys

Over the 4 weeks, learners had the opportunity to provide feedback on the level of engagement, usefulness, value, satisfaction and areas for improvement in the course content, through participation in weekly surveys. These surveys contained scales (strongly disagree to strongly agree) and asked questions like “how useful did you find this topic”; “how engaged did you feel”; and open boxes for free text responses. Descriptive statistics such as frequency, mean and standard deviation were used to summarise the data from these questions. Completion of these weekly surveys ranged from 17 to 20 learners each week (Supplementary Fig. 3).

#### Qualitative coding of free text responses

To analyse the text response according to our research questions, we first de-identified the transcripts for participant and institution names. The transcripts were uploaded to NVivo^®^ software for qualitative content analysis [16]. A coder analysed the transcripts and developed a series of codes. These codes were then used to categorise quotes, calculate frequencies and generate high level themes, in alignment with the research questions.

## Results

### Demographics

The pilot cohort consisted of *N* = 21 learners, *N* = 8 from the UK and *N* = 13 from Australia. Learners were from diverse healthcare professional backgrounds, including research and academia, tertiary care and private care. *N* = 21 learners completed the pre-survey (Week 0) (100% response rate) and *N* = 19 learners completed the post-survey (Week 12) (90% response rate). Four learners were lost to follow-up during the final week because they were ill, dropped out due to over commitment, or did not respond to requests.

From results of the pre-course survey, the level of previous experience with AI was variable, with the majority having no previous experience (*N* = 12/21) and some stating novice experience with AI (*N* = 7/21), and *N* = 2 with proficient experience. In terms of future applications of AI in their workplace, some learners were anticipating a future application (*N* = 6/21), others were still contemplating (*N* = 10/21) and a few were already implementing projects with AI (*N* = 5/21). Details around their industry or sector of work, previous experience with machine learning (ML) and AI, and how they plan to apply ML and AI into their workplace is detailed in Table 3.

**Table 3:** Demographics of the pilot course from Pre-course survey (*N* = 21). *Participants with several roles selected multiple sectors

Industry/sector	*N*	Previous experience with AI	*N*	Application of AI in workplace	*N*
Academia/Research	12	No previous experience	12	Anticipating a specific future implementation of AI	6
Tertiary Care	6	Novice/infrequent experience	7	Curious/Contemplating future implementation of AI	8
Specialist	6	Proficient	2	Currently implementing AI at my organisation	5
Healthcare technology	1	Expert/frequent user	0	Undecided whether to implement AI	2
Total Responses	*25		21		21

### How did learners’ self-efficacy in machine learning topics change after the course?

**Fig. 2:**
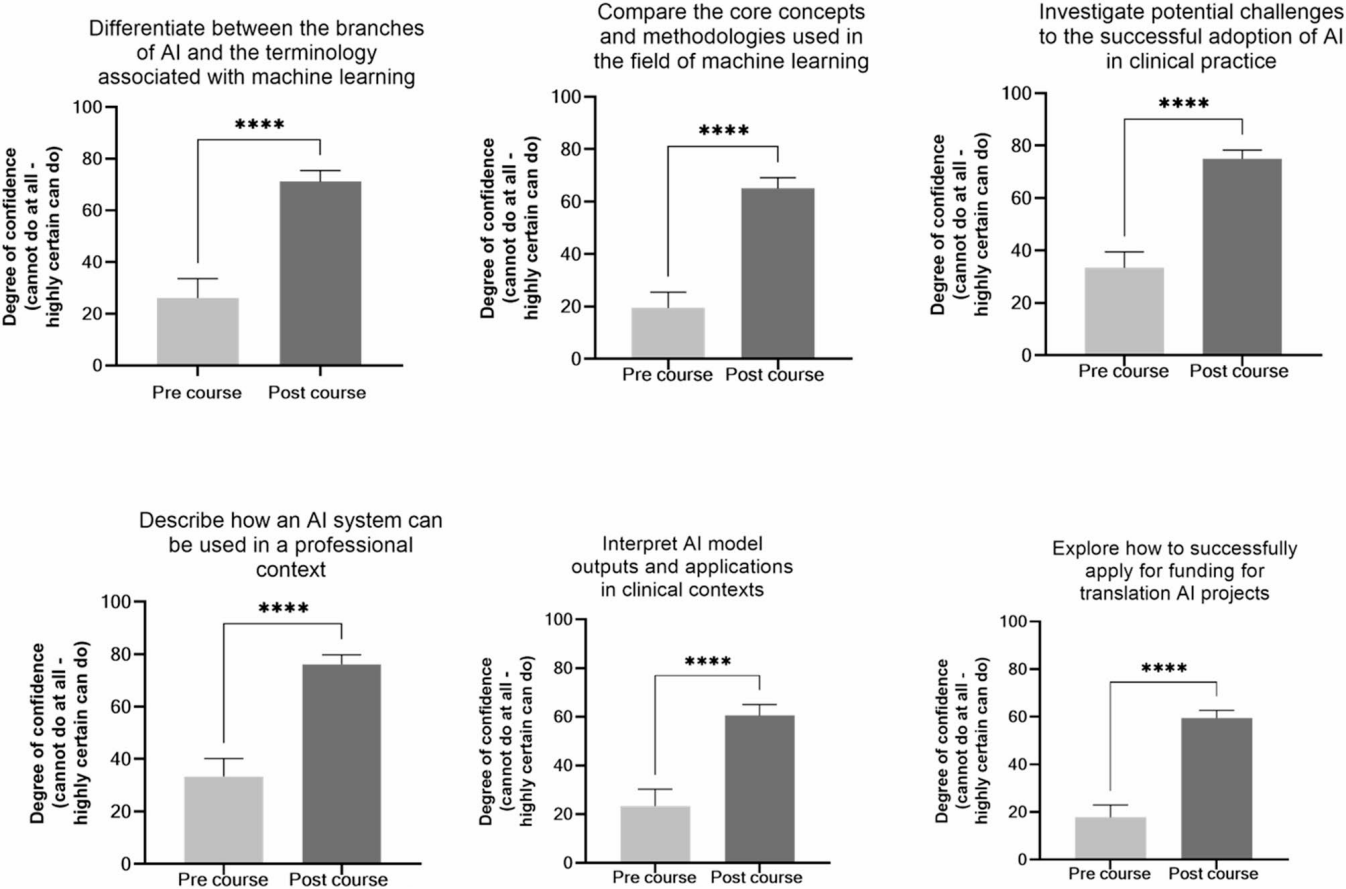
Pre course and post course self-efficacy rated against key machine learning capability statements. Learners (*N* = 17) rated confidence on a scale of 0-100 (0 = cannot do at all – 100 = highly certain can do). To compare the mean of the pre and post self-efficacy surveys, a two-tailed, paired t-test was undertaken (*N* = 21 pre-course, *N* = 17 post-course). Changes from baseline to post course confidence are shown for each learning outcome (*P* <.0001****)

To explore the change in self-confidence levels pre and post course, learners were surveyed on the key capability statements that the learning outcomes were mapped to. Learners completed the same set of ratings at the beginning and at the end of the course, following completion of all the material. For all learning outcomes, there was a statistically significant increase in self-efficacy as measured across all capability statements (*N* = 17, *P* <.0001) (Fig. 2).

### What type of teaching approaches did learners perceive as effective?

Given the number of diverse pedagogical approaches implemented in the course, we asked learners to rate the value of these various elements. The most highly rated was access to knowledgeable instructors, with all learners finding them either valuable or extremely valuable (100%): “*excellent expert panel and faculty; access to leaders in the field; global instructors and participants”*. This was followed by the self-paced online modules (95%) and synchronous workshop activities (89%): *“pre reading and in class learning works well*; *online classroom activities; interactive lessons and workshops”*, instructor feedback, the Topol panel: *“these were very informative”*, and case studies (83%), the pitchathon activity (78%): “*pitch presentation was an excellent exercise and allowed us to consider what had been covered in 4 weeks; as a concept*,* the final pitch is a good way to end the course”*, and lastly learning from colleagues (72%). In terms of applicability, highest rated were access to knowledgeable instructors (88%) and the Topol panel (78%), followed by the pitchathon (72%): *“pitchathon- it was good to use the knowledge gain to pitch an idea to specialists”* and self-paced online modules (72%), instructor feedback (67%), learning from colleagues (61%): “*international cohort and small group breakout session structure; discussion with previous fellows; workshopping with various experts in health”*, case studies (61%), and in-class live workshops (61%). Learners were asked to rate the degree of challenge and engagement with each topics pre-class learning and in-class sessions. The majority found the self-paced material (67%) and in class workshops (72%) moderately challenging, and 100% found the material at least moderately engaging. Overall, learners were satisfied with the quality of instructors (100%): *“very helpful group of expert instructors”*, enjoyed the global classroom experience and learning from international experts (94%) and colleagues (89%): “*access to breakout rooms to discuss individual projects”* and learners enjoyed the online learning platform (84%).

### How did learners plan to apply their learning in the future?

When learners were asked how they planned to use their learnings in their workplace, there were several potential applications that were suggested. The main themes were around ‘improving service and efficiency’ by leveraging AI for operational processes including planning and scheduling. Another theme was ‘predictive models’, particularly in the context of ML models for clinical decision support, identifying molecular targets and proteomics: *“help reduce the burden on clinicians especially with the boom in newer molecular targets in cancer care”.* Others suggested that the foundational knowledge would assist them in understanding what it takes and communicating with their teams: ”*understanding the basics of AI to think about possible use cases*,* allows us to get involved in the conversations and understand more about the team needed to work together on a project; “already applying in my interaction with data scientists on projects we are running using AI”*, as well as applying for grants, and that the networking would result in potential collaborators on these project: *“email contacts to add to my network”.*

One major concept that we often try to teach in machine learning and AI is that the quality of the output is only as good as the input and thus, the importance of having good data. This message appeared to translate well to the participants: *“consideration of what data to collect in the first place and the accuracy of this data; first step is identifying what the correct data is we could use in the future and what the best way is to get this happening”.*

Interestingly, while most participants understood and appreciated the flipped classroom format, there were still some participants that struggled to grasp the concept, wanting more time in class to “teach”, summarise and go over pre-class material questions: *“more teaching from the faculty; perhaps touch on a bit more on the pre class material; perhaps summary*,* q and a”*. One suggestion was that more quizzes were incorporated into the material, so that participants could check their understanding and areas of weakness: *“I like having little ‘tests’ at the end of each section*,* helps me know where I need to go over again*”.

Supplementary Table 1 displays participant responses to questions on satisfaction, engagement, value, applicability relating to pre-class materials and in-class sessions. The first relating to usefulness (extremely useless to extremely useful) and engagement (extremely unengaged to engaged) for each weekly topic [1–4]. Learners’ ratings of value pertaining to various domains: overall value to personal career development for all topics, applicability to current workplace role for all topics, overall satisfaction with quality of course, recommendation, instructors, and choice to revisit; and value of educational activities: instructors, Zoom workshops, online pre-class activities, collaborative learning, case scenarios, TOPOL panel and pitchathon.

### What were the perceived benefits of the programme?

Learners were asked to state the benefits of the programme. The major themes that arose were that learners enjoyed the structure of the course, including the flipped classroom model and the online learning environment (pre-class material): *“well paced and pleasingly fluid structure and flow”.* Learners appreciated having close access to international experts through the instructors, the Topol panel discussion and the pitchathon feedback panel: “*amazing expertise every week; it was great hearing from experts made week 2 make a lot more sense”*. They also enjoyed the peer learning aspect and hearing diverse perspectives and experiences, as well as the opportunities to connect and network with international colleagues and experts: *“the Topol presentations - these were very informative. Networking was good too*”. Furthermore, learners enjoyed the interactive learning during in-class sessions. This included the pitchathon: *“it was good to use the knowledge gain to pitch an idea to specialists”*,* “the pitch helps us crystalise what we’ve learnt in an applied setting”*, Topol panel: *“panel members presenting their work and the challenges they faced was extremely reassuring and helpful”*, breakout rooms: *“enjoying the breakout room structure for small group tasks/learning”*, machine learning playground: *“playground is great for understanding the algorithms*”.

### What changes or improvements would learners suggest to the short course?

As part of the evaluation, we asked learners to provide feedback on the aspects of the course that could be improved. While overall learners rated the pre-class material very highly, some felt that it was a steep jump and learning curve between the introductory fundamentals week and the second week on machine learning models, particularly in terms of technical language: *“face to face was a big jump from the pre-reading; very technical data language and approaches which I found difficult to understand without a background in this area”.* Learners were provided with a machine learning playground where they were able to manipulate metrics for various machine learning algorithms. While those with some prior knowledge enjoyed the activity, others wanted more guidance on the choosing the right features and what the optimal metrics were: *“different levels of experience of participants made perhaps the playground challenging. Not all had an informatics or data science background or were familiar with ML approaches which made this section fun for some*,* challenging for others”.* Moreover, while a few case studies were presented, learners wanted more case studies for a variety of applications and geographical contexts, that were simplified in terms of complexity but covered the process in greater detail: “*The case study used for a foundation level was somewhat difficult; teaching application of models in greater depth; more examples*,* real world examples*,* more anecdotal examples”.*

For the journal club activity, learners were given a paper that was an example of a poorly written paper so that there would be a lot to critique and discuss. However, for those that were less familiar with concepts and terminologies, this proved to be a challenging task to comprehend and digest: “d*ifficult to read the paper with so many unfamiliar terms. Felt a big jump from the pre-class learnings”.* Learners instead suggested using a best practice example or also reviewing an example of a good study for comparison: *“many of the experts seemed disappointed in the quality of the example ML research article used. It would have been helpful to have a well-designed ML review article as a comparator”.*

The University of Manchester was also running a course for the Topol Digital Fellowship Programme concurrently, in which many of fellows were working on AI related projects. Both programmes met together for a session which consisted of a panel of experts and there were also some Fellows that presented their projects to the learners. Learners stated that they found a lot of value in hearing from the experts and Fellows regarding the various challenges that they had faced in implementing projects: *“panel members presenting their work and the challenges they faced was extremely reassuring and helpful”.* They would have also liked to have more time to interact with the fellows and experts to dive deeper into projects and ask more questions: “*felt anti-climatic to design and present a pitch*,* then not receive any feedback; smaller groups*,* with more in depth feedback”*.

There was limited time available for the in-class sessions, due the short length of the course (4 weeks), and particularly due to the time difference between the countries (morning in UK, night in Australia). Learners frequently stated that they would have liked longer sessions to interact and network more with peers, experts and instructors during the course. They suggested more access to instructors in longer breakout rooms to ask questions and get feedback. Additionally, during the pitchathon activity, they highlighted that rather than group feedback, there would have been more value from breaking into smaller groups for the pitches and more time for one on one, individualised feedback from experts on each of their pitches: *“allow more time to discuss with the experts and an opportunity for questions/discussion”*,* “more in depth*,* individual feedback to our pitch*,* i.e. feedback by each expert after each pitch. may take longer and need 2 sessions maybe”.*

## Discussion

### Effectiveness of the flipped classroom model

The design of our global classroom was grounded in the flipped classroom model, underpinned by social [17] and active learning [18] pedagogical methods. By integrating self-paced asynchronous learning with interactive synchronous sessions, this model supports active and collaborative learning, making it especially suitable for time-poor healthcare professionals. Many of these professions also work in interprofessional teams, so this provides the opportunity to learn from and share experiences with one another. This model enabled learners to explore foundational concepts at their own pace and then apply them during interactive discussions and practical activities. By using this approach, we aimed to support learners from different global time zones, who may be managing demanding schedules around their studies. We drew on Laurillard’s conversational framework [17] which views learning as an iterative and incremental process, to ensure there were enough opportunities for learners to build and refine their understanding of machine learning through the combination of self-led investigative activities and group discussions.

### Learner engagement, motivation and satisfaction

From the results of the evaluation, it is clear; the learners enjoyed the structure of the course, the combination of pre-class materials and online activities. Our online global classroom provided a “good taster” on the topic of machine learning in healthcare; however, there are some potential areas of improvement to consider for future cohorts. For example, the feedback illustrates some learners found there was a jump in the depth and pace of the content, leading to a steep learning curve for many. This is understandable, given the diverse roles, abilities, and experiences of AI in healthcare. This feedback suggests a need for a more scaffolded approach to content delivery, perhaps through differentiated learning pathways tailored to varied technical proficiency levels. Additionally, more in-depth reading materials could be provided for those that wanted to deep dive further into topics. The cohort asked for more time for small group learning and self-reflection which aligns with the features found in other global classroom environments [5]. The learners reported they found flexibility of online synchronous and asynchronous activities the most beneficial elements of the short course. However, more support, and context specific examples, case studies and customisation of the content, could potentially help learners apply concepts to practice in a more applied way. This would also accommodate for diverse learner backgrounds and experience levels.

### Impact on professional practice and long-term application

It is clear the course raised awareness of machine learning in healthcare, as well as increasing confidence and motivation across all capabilities, illustrating a desire to develop knowledge further. However, due to the short-term nature of global professional development courses, such as this, we have not been able to evidence how this would impact in the longer term. This behavioural change, as set out in the Kirkpatrick framework [12] requires continuing self-determined application of learning to practice. Further pedagogic research in the form of follow up interviews with the cohort will need to be undertaken to determine how participants utilised the learnings and if there were any meaningful changes to their practice. Additionally, creating opportunities for sustained interaction beyond the course, such as mentoring programmes or collaborative projects, could help build a global community of practice in digital health technology.

### Global classroom reflections

The collaborative nature of this initiative was a core strength, allowing participants to connect across institutions in the United Kingdom and Australia. Participants rated various components, such as live workshops, expert panels, pitchathon, and peer-learning activities, as valuable. Access to knowledgeable instructors and opportunities for real-time interaction were particularly well-received, which demonstrated the importance of involving domain experts to contextualise complex topics like ML. However, the desire for more in-depth discussions and networking opportunities indicates a need to expand the duration or re-structure synchronous sessions to allow for more individualised feedback. Learners reported gaining diverse perspectives and practical insights, reinforcing the importance of interdisciplinary collaboration in addressing healthcare challenges, also noted by Bailey et al. [19]. Additional support mechanisms, such as extended access to mentors or peer groups, could further sustain motivation and long-term skill development. The ability of the course to foster an international network of healthcare professionals is a significant outcome, but there is scope to further enhance these networks through structured communities of practice that extend beyond the course duration.

We wanted to recognise and reflect on the different perspectives from both countries and broaden the network for our learners. Whilst students have typically learned global transdisciplinary experiences via study abroad programmes - this is often cost prohibitive for some programmes, thus us driving collaborative online international learning (COIL) as a model for global knowledge exchange. The wealth of available technologies (Teams, Zoom, virtual learning environments) allows students and educators to connect in different countries and work on real world problems that they can share using such technology.

Unlike other global classrooms often used in undergraduate teaching, our group all were qualified professionals, most with many years of experience working in their respective careers. Therefore, the motivations of our learners for joining this course were likely very different from the undergraduate setting, which might be more focussed on working collaboratively on projects to learn more about cross-cultural issues, for example. Our pre-course survey data illustrated that many learners were motivated by their curiosity or consideration in implementing a future AI based application, as well as gaining exposure to health system differences [19] and how other countries approach these problems [8].

### Learnings from international educational collaboration

The benefit of international collaboration is the sharing of learnings, and the adoption of aspects that worked well between each institution. As an international team we were able to learn a lot from each other, with the Melbourne team having more experience of delivering more blended and flipped classroom models, some of which we adopted within this programme - including the final pitchathon session. As noted by Carroll et al., regarding the TEaCh initiative (a global classroom between three universities in Ireland and USA for public health teaching), we also needed to recognise and incorporate the cultural and structural differences of the two educational teams involved in developing the programme [5]. The Manchester team had existing materials related to AI and machine learning and a rich source of translational case studies and subject matter experts they could draw on to support the development and delivery of the course content. This is likely reflective of the more wide-spread adoption of electronic health records in the NHS, and the associated capture and use of data for translational research, thus driving the development of machine learning algorithms.

The process of co-creation was supported by synchronous meetings (held monthly over a period of 4 months with an additional face to face meeting between course leads at the University of Melbourne, which was good to affirm the collaboration and knowledge exchange, a success factor also noted by Caroll et al. Given the difficult timing of collaborative meetings given the time zone difference, all educators had to buy into the shared vision and the potential outcomes of the collaboration. The authors also noted the need for a collective commitment to making their collaboration work on limited funding, a sentiment we also shared, noting a very modest investment to support our course development. We developed a joint vision and enthusiasm for learning and evolving our practice, a willingness to share resources between institutions and to attend meetings in a regular manner to build the relationships within the partnership. As also noted by Caroll et al., levels of learner engagement were high with good attendance at synchronous sessions. Fifteen participants of the cohort presented at the final pitch event, which may also have been influenced by the fact that learners were aware they were presenting to international colleagues. Additionally, we made participation a required commitment to gain access to the free pilot. Moreover, building towards the final pitch was scaffold throughout the weeks with various prompting questions that built up to the final output.

### Limitations and constraints

The small sample size and reliance on self-reported data, may affect the generalisability of findings. The short course duration also limited insights into the long-term impacts of the programme on participants’ professional practice. Future research could involve longitudinal studies to track the application of course learnings over time and explore the potential for scaling this model to larger global classroom cohorts and other interdisciplinary topics. Additionally, while this pilot was free, in order to test feasibility, future models would need to investigate licensing agreements and arrangements for profit share models between institutions, to ensure long-term sustainability of the program.

Other logistical challenges included the timing of synchronous sessions to factor in both time zones and clinical commitments (for example, too early in the day would likely also clash with clinic times, whilst lunch time to coincide with time away from clinics would only work for one half of the cohort). Use of our E-lab environment to host learning materials (supported in Articulate Rise) and also Flask apps to enable the learners to try the algorithms, negated the need to register the learners as students on our virtual learning environment and helped to reduce the admin and barriers to accessing course materials. Links to all synchronous sessions were also provided within eLab, and we avoided use of resources that were only accessible via the University libraries. Though we recognise in the future this might be a limiting factor as we may require the learners to access relevant journal articles and literature to support their learning.

From a learner perspective some of the main challenges when working in a clinical setting will revolve around protected study time to undertake the professional learning, especially with clinical rotas often being set so far in advance. In addition, these pressures are also likely to impact on the learners’ capacity to implement any further changes to practice (which might impact our Kirkpatrick level 3 and 4 evaluations – changes to behaviour and changes to practice.) In order to see longer term changes in both behaviours and practices a more involved programme akin to the Australian LHS Academy [20], UK Topol Fellowship programme [10] or Clinical AI Fellowship programmes may be required [21]. These programmes release healthcare professionals from clinical practice, with protected study time, and will likely therefore provide further opportunities for implementation of their AI ideas into practice.

On reflection our learning outcomes were likely overly ambitious with such a short course. This including learning outcomes centred on higher level blooms taxonomy including “interpret” and “investigate”, which was perhaps a stretch for the time available to our learners.

### Future of the program.

A year is a long time in this area, things are changing all the time, skills and capabilities are developing, new roles emerging, and AI-enabled approaches and scale of technology continues to proliferate at pace. Ideally, we would like to follow the career trajectory of our learners, over the medium to long term period, through initiatives such as a fellowship-style approach, with opportunities for cross-institutional travel to share and bring back learnings. However, in the short term, we will explore the following improvements to our global classroom:

- Personalisation of learning content, to help learners choose the level of technical machine learning content they want to learn about.
- Enhancing critical evaluation of ML activities by adding more time for reflection, be it on an individual basis or within groups, based on geography or area of interest.
- To obtain a truly global perspective and build on success of including the Topol Fellows, by inviting a broader variety of views and experiences, including more patient or public inclusion and/or local community groups.
- Offer more formalised long term collaborative learning activities, during and post course, such as, through mentoring, project-based opportunities to build communities of practice (a resource intensive endeavour).

Areas for investigation to provide more collaborations amongst the teaching team include:

- Work on other projects related to digital health education, for example through educational tools or games, relevant case studies, networking through community of practices in both countries.
- To explore capabilities at an international level and create frameworks and evaluation tools for others to adopt in their practice.
- Longer term collaborations such as secondments and other activities, to help build a global digital health education community of practice.

## Conclusion

This programme demonstrated the effectiveness of the global classroom delivery strategy in the rapidly evolving AI/ML space. Health professionals internationally will undoubtedly need to be upskilled in this new discipline and thus utilising the global classroom method may be an effective pedagogical strategy, while also reducing replication of effort, and consolidation and sharing of resources (particularly case studies) between institutions. It also provides an opportunity for academics to learn from the practices of each institution and take on valuable peer feedback and scholarship. For international learners, the global classroom offers opportunities to learn from and share learnings and experiences of different health systems and digital transformation projects, across institutions. It also provides access to international expertise for knowledge sharing, problem solving and collaboration efforts. There are significant opportunities to grow these efforts into ongoing collaborative communities of practice. In spite of logistical challenges of time zones, and need for careful consideration of sustainability efforts, this programme may prove to be a useful prototype for other educational institutions to adopt aspects and tailor them to various fields.

## Supplementary Information

Supplementary Material 1.

## Data Availability

Data is provided within the manuscript or supplementary information files.
