# Supplementary material for: Evaluating a global classroom initiative to teach machine learning applications in healthcare
Source: BMC Med Educ. 2025 Oct 21;25:1455. doi: 10.1186/s12909-025-07918-w (PMC12538724; doi:10.1186/s12909-025-07918-w)
Supplement: Supplementary file 1 — Supplementary Material 1. [file 12909_2025_7918_MOESM1_ESM.docx]

## **Supplementary material**

#### Supplementary Table 1. Feedback on pre-class learning materials and in-class zoom sessions.

| **Rating** | **Very unsatisfied** | | **Unsatisfied** | | **Neutral** | | **Satisfied** | | **Very satisfied** | | **TOTAL** | |
| --- | --- | --- | --- | --- | --- | --- | --- | --- | --- | --- | --- | --- |
| **Question** – **Pre-class materials satisfaction with modules** | **%** | **Freq** | **%** | **Freq** | **%** | **Freq** | **%** | **Freq** | **%** | **Freq** | **Total Freq** | |
| The fundamentals of machine learning |  |  |  |  | 5 | 1 | 28 | 5 | 67 | 12 | 18 | |
| Machine learning models |  |  |  |  |  |  | 44 | 8 | 56 | 10 | 18 | |
| Trust in machine learning outputs |  |  |  |  | 22 | 4 | 56 | 10 | 22 | 4 | 18 | |
| Considerations for AI use in healthcare |  |  |  |  | 22 | 4 | 33 | 6 | 44 | 8 | 18 | |
| **Question** – **In-class session satisfaction with activities** | **%** | **Freq** | **%** | **Freq** | **%** | **Freq** | **%** | **Freq** | **%** | **Freq** | **Total Freq** | |
| The fundamentals of machine learning |  |  |  |  | 17 | 3 | 33 | 6 | 50 | 9 | 18 | |
| Machine learning models |  |  | 6 | 1 | 6 | 1 | 44 | 8 | 44 | 8 | 18 | |
| Trust in machine learning outputs |  |  |  |  | 17 | 3 | 39 | 7 | 44 | 8 | 18 | |
| Considerations for AI use in healthcare |  |  | 6 | 1 | 11 | 2 | 50 | 9 | 33 | 6 | 18 | |
| **Rating** | **Not challenging** | | **Slightly challenging** | | **Moderately challenging** | | **Very challenging** | | **Extremely challenging** | | **TOTAL** | |
| **Question – Degree of challenge** | **%** | **Freq** | **%** | **Freq** | **%** | **Freq** | **%** | **Freq** | **%** | **Freq** | **Total Freq** | |
| Self-paced online modules |  |  | 22 | 4 | 67 | 12 | 5.5 | 1 | 5.5 | 1 | 18 | |
| In-class live workshop activities |  |  | 17 | 3 | 72 | 13 |  |  | 11 | 2 | 18 | |
| **Rating** | **Not engaging** | | **Slightly engaging** | | **Moderately engaging** | | **Very engaging** | | **Extremely engaging** | | **TOTAL** | |
| **Question – Degree of engagement** | **%** | **Freq** | **%** | **Freq** | **%** | **Freq** | **%** | **Freq** | **%** | **Freq** | **Total Freq** | |
| Self-paced online modules |  |  |  |  | 39 | 7 | 50 | 9 | 11 | 2 | 18 | |
| In-class live workshop activities |  |  |  |  | 39 | 7 | 50 | 9 | 11 | 2 | 18 | |
| **Rating** | **Highly unvaluable** | | **Unvaluable** | | **Neutral** | | **Valuable** | | **Highly valuable** | | **TOTAL** |  |
| **Question** - Valuable to your personal career development (N=18) | **%** | **Freq** | **%** | **Freq** | **%** | **Freq** | **%** | **Freq** | **%** | **Freq** | **Total Freq** | |
| Access to a knowledgeable instructor |  |  |  |  |  |  | 33 | 6 | 67 | 12 | 18 | |
| Self-paced online modules |  |  | 6 | 1 |  |  | 39 | 7 | 56 | 10 | 18 | |
| Learning with and from my colleagues |  |  | 6 | 1 | 22 | 4 | 28 | 5 | 44 | 8 | 18 | |
| Instructor feedback |  |  | 6 | 1 | 11 | 2 | 44 | 8 | 39 | 7 | 18 | |
| Case studies |  |  |  |  | 17 | 3 | 44 | 8 | 39 | 7 | 18 | |
| The TOPOL Panel |  |  | 6 | 1 | 11 | 2 | 44 | 8 | 39 | 7 | 18 | |
| Pitchathon Activity |  |  | 5 | 1 | 17 | 3 | 39 | 7 | 39 | 7 | 18 | |
| In-class live workshop activities |  |  |  |  | 11 | 2 | 56 | 10 | 33 | 6 | 18 | |
| **Rating** | **Highly not applicable** | | **Not applicable** | | **Neutral** | | **Applicable** | | **Highly applicable** | | **TOTAL** | |
| **Question -** Applicability to your current workplace role (N=18) | **%** | **Freq** | **%** | **Freq** | **%** | **Freq** | **%** | **Freq** | **%** | **Freq** | **Total Freq** | |
| Access to a knowledgeable instructor |  |  | 6 | 1 | 6 | 1 | 44 | 8 | 44 | 8 | 18 | |
| Self-paced online modules |  |  | 6 | 1 | 22 | 4 | 44 | 8 | 28 | 5 | 18 | |
| Learning with and from my colleagues |  |  | 11 | 2 | 28 | 5 | 22 | 4 | 39 | 7 | 18 | |
| Instructor feedback |  |  | 17 | 3 | 17 | 3 | 50 | 9 | 17 | 3 | 18 | |
| Case studies |  |  | 17 | 3 | 22 | 4 | 28 | 5 | 33 | 6 | 18 | |
| The TOPOL Panel |  |  | 5 | 1 | 17 | 3 | 61 | 11 | 17 | 3 | 18 | |
| Pitchathon Activity |  |  | 6 | 1 | 22 | 4 | 44 | 8 | 28 | 5 | 18 | |
| In-class live workshop activities |  |  | 6 | 1 | 33 | 6 | 33 | 6 | 28 | 5 | 18 | |
| **Rating** | **Extremely valueless** | | **Moderately valueless** | | **Neither valuable nor valueless** | | **Slightly valuable** | | **Highly valuable** | | **TOTAL** | |
| **Questions –** Agreement with the following statements | **%** | **Freq** | **%** | **Freq** | **%** | **Freq** | **%** | **Freq** | **%** | **Freq** | **Total Freq** | |
| Overall, I am satisfied with the quality of instructors |  |  |  |  |  |  | 39 | 7 | 61 | 11 | 17 | |
| If I could revisit the choice to complete the course |  |  |  |  | 11 | 1 | 28 | 5 | 61 | 11 | 17 | |
| Overall, I enjoyed the global classroom, learning from international experts |  |  |  |  |  |  | 33 | 6 | 61 | 11 | 17 | |
| Overall, I enjoyed the global classroom, learning alongside colleagues |  |  |  |  | 11 | 1 | 33 | 6 | 56 | 10 | 17 | |
| Overall, I enjoyed the online learning platform |  |  |  |  | 16 | 2 | 28 | 5 | 56 | 10 | 17 | |
| I would recommend the course to a colleague or my team |  |  |  |  | 11 | 1 | 39 | 7 | 50 | 9 | 17 | |
| Overall the course was useful to my work |  |  |  |  | 11 | 1 | 61 | 11 | 33 | 6 | 17 | |
| Overall, the course was applicable to my work |  |  |  |  | 23 | 2 | 44 | 8 | 33 | 6 | 16 | |
| Overall, I am satisfied with the quality of the course |  |  |  |  |  |  | 61 | 11 | 33 | 6 | 17 | |
| Overall, the course was valuable to my work |  |  |  |  | 11 | 1 | 61 | 11 | 28 | 5 | 17 | |

|  |
| --- |

### **Supplementary Figure 1 - Pre Course Survey**

Q As part of enrolling in this free course, I agree to complete the following by checking each box:

***If you cannot agree to any of these, we will offer your spot to someone else. Please email us at sathana.dushyanthen@unimelb.edu.au to notify us that you cannot agree to any/one of these items or have any questions.*

- Complete 3 hours of online self-directed modules a week for four weeks (prior to the Zoom virtual workshops)
- Attend all of the virtual (Zoom) workshops on 25th of Sept, 2nd of Oct, 9th of Oct, and 16th of Oct
- Be ready to fully engage in the Zoom workshops
- Complete a 20 minute post-survey after the course
- Participate in workshops with your web CAMERA ON (unless you have extenuating circumstances, e.g., joining from a clinical environment)

Q The following lists the learning goals for the course. Rate how confident you are that you can do them as of now. Rate your degree of confidence by recording a number from 0 to 100 using the scale given below:

**NB. If you choose '0' you must click on '0' to register the response.**

|  | Cannot do at all | Moderately certain can do | Highly certain can do |
| --- | --- | --- | --- |

|  | 0 | 10 | 20 | 30 | 40 | 50 | 60 | 70 | 80 | 90 | 100 |
| --- | --- | --- | --- | --- | --- | --- | --- | --- | --- | --- | --- |

| Identify potential machine learning applications in my workplace or research () | 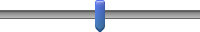 |
| --- | --- |
| Differentiate between the branches of AI and the terminology associated with machine learning () | 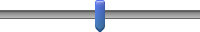 |
| Compare the core concepts and methodologies used in the field of machine learning (e.g. data science, statistics, mathematics, computer programming) () | 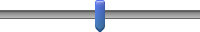 |
| Investigate potential challenges to the successful adoption of AI in clinical practice () | 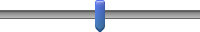 |
| Describe how an AI system can be used in a professional context () | 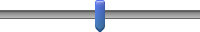 |
| Interpret AI model outputs and applications in clinical contexts () | 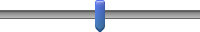 |
| Explore how to successfully apply for funding for translation AI projects () | 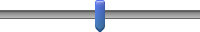 |

### **Supplementary Figure 2 - Post-course survey**

Q3 What is your current level of involvement in an AI project or application?

- Currently implementing AI at my organisation (1)
- Anticipating a specific future implementation of AI (2)
- Curious / Contemplating future implementation of AI (3)
- Undecided of whether to implement AI (4)
- Other (5) __________________________________________________

Q4 For the following educational activities, complete the following ratings:

|  | Valuable to your personal career development | | | | | Applicability to your current workplace role | | | | |
| --- | --- | --- | --- | --- | --- | --- | --- | --- | --- | --- |
|  | Highly unvaluable (1) | Unvaluable (2) | Neutral (3) | Valuable (4) | Highly valuable (5) | Highly not applicable (1) | Not applicable (2) | Neutral (3) | Applicable (4) | Highly Applicable (5) |
| The self-paced online modules (1) |  |  |  |  |  |  |  |  |  |  |
| The in-class live workshop activities (2) |  |  |  |  |  |  |  |  |  |  |
| Access to a knowledgeable instructor (3) |  |  |  |  |  |  |  |  |  |  |
| Instructor feedback (4) |  |  |  |  |  |  |  |  |  |  |
| Learning with and from my colleagues in the course (5) |  |  |  |  |  |  |  |  |  |  |
| Case studies (9) |  |  |  |  |  |  |  |  |  |  |
| The TOPOL panlel (7) |  |  |  |  |  |  |  |  |  |  |
| The Pitchathon activity (8) |  |  |  |  |  |  |  |  |  |  |
| Other, please state (6) |  |  |  |  |  |  |  |  |  |  |

Q5 For the following educational activities, complete the following ratings:

|  | Degree of challenge | | | | | Degree of engagement | | | | |
| --- | --- | --- | --- | --- | --- | --- | --- | --- | --- | --- |
|  | Not challenging at all (1) | Slightly challenging (2) | Moderately challenging (3) | Very challenging (4) | Extremely challenging (5) | Not engaging (1) | Slightly engaging (2) | Moderately engaging (3) | Very engaging (4) | Extremely engaging (5) |
| The self-paced online modules (1) |  |  |  |  |  |  |  |  |  |  |
| The in-class live workshop activities (2) |  |  |  |  |  |  |  |  |  |  |

Q6 For the following educational activities, complete the following ratings:

|  | Pre-class | | | | | In-class | | | | | |
| --- | --- | --- | --- | --- | --- | --- | --- | --- | --- | --- | --- |
|  | Very unsatisfied (1) | Unsatisfied (2) | Neutral (3) | Satisfied (4) | Very Satisfied (5) | Very unsatisfied (1) | Unsatisfied (2) | Neutral (3) | Satisfied (4) | Very Satisfied (5) |  |
| Topic 1 - The fundamentals of machine learning (1) |  |  |  |  |  |  |  |  |  |  |  |
| Topic 2 - Machine learning models (2) |  |  |  |  |  |  |  |  |  |  |  |
| Topic 3 - Trust in machine learning outputs [Topol panel] (7) |  |  |  |  |  |  |  |  |  |  |  |
| Topic 4 - Considerations for AI use in healthcare [Pitchathon] (8) |  |  |  |  |  |  |  |  |  |  |  |

Q7 Please indicate the extent to which you agree with the following statements:

|  | Strongly disagree (1) | Somewhat disagree (2) | Neither agree nor disagree (3) | Somewhat agree (4) | Strongly agree (5) |
| --- | --- | --- | --- | --- | --- |
| Overall, the course was useful to my work (1) |  |  |  |  |  |
| Overall, the course as valuable to my work (2) |  |  |  |  |  |
| Overall, the course was applicable to my work (3) |  |  |  |  |  |
| Overall, I am satisfied with the quality of the course (4) |  |  |  |  |  |
| Overall, I am satisfied with the quality of the instructors (5) |  |  |  |  |  |
| Overall, I enjoyed the global classroom, learning alongside colleagues (8) |  |  |  |  |  |
| Overall, I enjoyed the global classroom, learning from international experts (9) |  |  |  |  |  |
| Overall, I enjoyed the online learning platform (10) |  |  |  |  |  |
| If I could revisit the choice to complete this course, then I would choose to complete it again (6) |  |  |  |  |  |
| I would recommend the course to a colleague or my team (7) |  |  |  |  |  |

Q8 On average, how much time did you regularly spend outside of the Zoom sessions engaging with the material (i.e., pre-class materials)?

- Less than one hour (1)
- One hour (2)
- Two hours (3)
- Three hours (4)
- More than three hours, indicate here: (5)

Q9 Overall, how much did you learn from the course?

- None at all (1)
- A little (2)
- A moderate amount (3)
- A lot (4)
- A great deal (5)

Q10 The following lists the learning goals for the course.

Rate how confident you are that you can do them as of now. Rate your degree of confidence by recording a number from 0 to 100 using the scale given below:

**NB. If you choose '0' you must click on '0' to register the response.**

|  | Cannot do at all | Moderately certain can do | Highly certain can do |
| --- | --- | --- | --- |

|  | 0 | 10 | 20 | 30 | 40 | 50 | 60 | 70 | 80 | 90 | 100 |
| --- | --- | --- | --- | --- | --- | --- | --- | --- | --- | --- | --- |

| Differentiate between the branches of AI and the terminology associated with machine learning () | 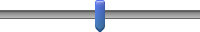 |
| --- | --- |
| Compare the core concepts and methodologies used in the field of machine learning (e.g. data science, statistics, mathematics, computer programming) () | 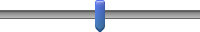 |
| Investigate potential challenges to the successful adoption of AI in clinical practice (e.g. accessing data, data quality, ethics and governance) () | 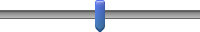 |
| Describe how an AI system can be used in a professional context () | 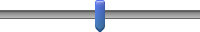 |
| Interpret AI model outputs and applications in clinical contexts () | 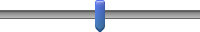 |
| Explore how to successfully apply for funding for translation AI projects () | 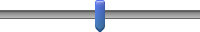 |

| Page Break |  |
| --- | --- |

Q11 What aspects of the course should we KEEP?

________________________________________________________________

Q12 What aspects of the course should we CHANGE (START/STOP)?

________________________________________________________________

Q13 What were the most beneficial elements of the short course?

________________________________________________________________

Q14 Beyond what you have written in weekly surveys, what would you improve of the course for future iterations? e.g., topics, activities, instructors, delivery, structure, etc.

________________________________________________________________

Q15 How do you expect to apply what you learn in the course to your current workplace? Or future workplace?

________________________________________________________________

Q16 If we created more AI related courses, what types of topics or courses would you want to take?

________________________________________________________________

Q17 Are you interested in networking in the future and if so what type activity would you find helpful?

- Access to relevant listservs or emails (1)
- Access to shared bookmarked resources (2)
- Notification of future session events being run by both Universities (3)
- Notification of credit bearing courses and case studies (4)
- Other (please specify) (5) __________________________________________________

Q18 If you have any other comments, please share them here:

________________________________________________________________

Supplementary Figure 3. Weekly feedback surveys.

Q1 Which topic are you evaluating?
*please submit a new survey for each topic*

▼ Topic 1 - The fundamentals of machine learning (3) ... Topic 4 - Considerations for the use of AI in healthcare (6)

Q2  Rate the extent to which you agree with the following statements:

|  | Extremely unsatisfied or unengaged (1) | Unsatisfied or unengaged (2) | Neutral (4) | Satisfied or engaged (6) | Extremely satisfied or engaged (7) |
| --- | --- | --- | --- | --- | --- |
| "I found this topic's pre-class learning useful". (1) |  |  |  |  |  |
| "I found this topic's in-class session useful". (2) |  |  |  |  |  |
| "I felt engaged when completing the pre-class learning for this topic". (3) |  |  |  |  |  |
| "I felt engaged when participating in the topic's in-class session". (4) |  |  |  |  |  |

Q3 I recommend to KEEP the following aspects:

________________________________________________________________

Q4 I recommend to CHANGE the following aspects:

________________________________________________________________

Q5 Are there any other comments, feedback, or suggestions that you would like to provide?

________________________________________________________________
